# Alcohol Use among Adolescent Youth: The Role of Friendship Networks and Family Factors in Multiple School Studies

**DOI:** 10.1371/journal.pone.0119965

**Published:** 2015-03-10

**Authors:** Cheng Wang, John R. Hipp, Carter T. Butts, Rupa Jose, Cynthia M. Lakon

**Affiliations:** 1 Department of Sociology, Cornell University, Ithaca, New York, United States of America; 2 Departments of Criminology, Law and Society and Sociology, University of California Irvine, Irvine, California, United States of America; 3 Departments of Sociology and Statistics, University of California Irvine, Irvine, California, United States of America; 4 Department of Psychology and Social Behavior, University of California Irvine, Irvine, California, United States of America; 5 Program in Public Health, University of California Irvine, Irvine, California, United States of America; David Geffen School of Medicine at UCLA, UNITED STATES

## Abstract

To explore the co-evolution of friendship tie choice and alcohol use behavior among 1,284 adolescents from 12 small schools and 976 adolescents from one big school sampled in the National Longitudinal Study of Adolescent to Adult Health (AddHealth), we apply a Stochastic Actor-Based (SAB) approach implemented in the R-based Simulation Investigation for Empirical Network Analysis (RSiena) package. Our results indicate the salience of both peer selection and peer influence effects for friendship tie choice and adolescent drinking behavior. Concurrently, the main effect models indicate that parental monitoring and the parental home drinking environment affected adolescent alcohol use in the small school sample, and that parental home drinking environment affected adolescent drinking in the large school sample. In the small school sample, we detect an interaction between the parental home drinking environment and choosing friends that drink as they multiplicatively affect friendship tie choice. Our findings suggest that future research should investigate the synergistic effects of both peer and parental influences for adolescent friendship tie choices and drinking behavior. And given the tendency of adolescents to form ties with their friends' friends, and the evidence of local hierarchy in these networks, popular youth who do not drink may be uniquely positioned and uniquely salient as the highest rank of the hierarchy to cause anti-drinking peer influences to diffuse down the social hierarchy to less popular youth. As such, future interventions should harness prosocial peer influences simultaneously with strategies to increase parental support and monitoring among parents to promote affiliation with prosocial peers.

## Introduction

In the United States, the prevalence of adolescent drinking has declined since the late 1990s—however the problem is far from being solved. Recent reports find that 71% of 9th- to 12th-grade students indicate having had at least one drink of alcohol (other than a few sips) during their lifetime, with about one fifth of them starting to drink by age 13 [1]. The high prevalence of underage drinkers, and early initiation into drinking practices, has led to decades of research on the consequences of adolescent alcohol use. Studies have linked adolescent drinking to other adolescent problem behaviors, including automobile accidents [2,3,4,6], drug abuse [3,4,8], engagement in risky sexual activities [2,3,4], school absenteeism [3,9], and poor or failing grades [3,4,5,7]. Thus, despite the declining rates of alcohol use among youth, adolescent drinking remains a key agenda item for the public health community to address.

Ecological models [10,11] suggest that multiple contextual influences shape adolescent development and health behavior. Adolescence is a critical time period during which various contextual influences—including those exerted by youths' friends and parents prominently as the primary socialization forces—affecting adolescent development and their health behavior. Theoretical intuition from ecological models suggests that both peer and parental influences might act both independently and in synergy as they impact the simultaneous processes of adolescent friendship tie choice and adolescent drinking.

Peer networks are a salient reference group for youth, with peers playing a particularly important and influential role in helping shape adolescents' evolving social worlds [12]. Various characteristics of adolescent peer networks are salient for youths' friendship tie choices. Reciprocity among adolescent friendship pairs is a strong force affecting friendship tie formation among adolescent youth [13]. Indicators of degree, including in-degree centrality which is the popularity of an actor in a network [14], and in-in degree assortativity, which is the tendency to choose network actors who are similarly popular, are important for friendship tie selection, as popular youth may have many options for forming friendships, and likewise may be more likely to choose similarly popular friends. Another degree based network indicator, out-degree, is an indicator of the number of ties a network actor sends [14], and reflects an actor's expansiveness in the network. Adolescents who send out more friendship tie nominations are salient in a network. In addition, the number of transitive triplets and three-cycles, which reflect local hierarchy in a network, are likely important for friendship tie choice as youth occupying positions in these triadic structures have an opportunity to influence one another, likely on multiple dimensions including on friendship tie choices.

In addition to shaping adolescents' friendship tie choices, peer networks also shape adolescent drinking behavior. Two important socialization processes operating in peer networks are peer influence and peer selection. Peer influence is the process wherein youth alter their behavior such that it aligns with that of their peers. Peer selection is the process by which youth select friends who are similar to themselves, on various dimensions. Adolescent alcohol use can be conceptualized as a result of both peer influence and selection processes, with friends influencing one another's drinking behavior and adolescents selecting friends who engage in similar levels of drinking [15–20]. And in-degree popularity has also been positively related to adolescent drinking behavior across studies [21–24].

Stochastic Actor-Based (SAB) models [25,26] have offered keen insights into adolescent alcohol use, allowing researchers to disentangle peer selection and peer influence effects. These dynamic models estimate friendship tie choice and drinking behavior changes simultaneously. One study found both selection (i.e., "birds of a feather" or homophily effects) and influence (i.e., social contagion) effects for drinking behavior when using longitudinal data of youth in the West of Scotland [27]. Similar studies based on longitudinal data collected in Netherlands [28], Italy [29], Finland [30], and Sweden [31] also found both selection and influence processes underlying adolescent drinking behavior similarity. The first study to utilize the SAB modeling strategy to examine the drinking behavior among adolescents in the United States was Mundt, Mercken, and Zakletskaia [32], using two waves of data from the National Longitudinal Study of Adolescent to Adult Health (AddHealth), a nationally representative survey of US adolescents from 7th to 12th grades between 1995 and 1996. Contrary to the findings from international studies, Mundt, Mercken, and Zakletskaia [32] found only a significant selection effect but no influence effect for alcohol use. A study utilizing data from 450 15-to-17 year-old students attending a public high school in the Northeastern United States between 2004 and 2006 showed the opposite effect, with only a significant peer influence effect on alcohol use [33]. Moreover, a third study, using the PROSPER dataset—which followed 13,214 6th-to-9th grade adolescents from 50 classrooms in Iowa and Pennsylvania during the fall of 2002 and 2003—reported both significant influence and selection effects for drinking behavior similarity [34].

Parents are also influential referents in adolescents' social worlds, affecting both adolescent friendship tie choices and their drinking behavior. Parents exert influences on the socialization of adolescent youth through multiple processes, including the provision of social support and parental monitoring. Indeed, parental influences have a critical impact on adolescent development, particularly in the area of building youths' social competence regarding friendship formation. One study indicated that familial factors are associated with social competence, peer acceptance, and the ability to form and maintain close friendships among youth [35]. Moreover, theoretical guidance from Social Control Theory [36,37] casts parental monitoring as a key process deterring youth from affiliating with delinquent peers, which may then act to lessen the probability that youth begin using substances such as alcohol. As such, parents may affect the likelihood that their adolescent children will select friends who drink. A recent cross-sectional study examining adolescent substance use suggested that youth reared in families characterized by a lack of familial obligations, emotional closeness, and support, were more likely to affiliate with substance using peers, and that moreover, having these peer relationships was associated with more substance use [38]. To the extent that parents are successful at monitoring their children, the adolescent child may have less opportunity to associate with friends who drink, as parents may prohibit friendships with other youth who drink or prohibit their adolescent children from being in situations which present opportunities to drink [39]. It is therefore possible that parents' characteristics might interact with youths' friendship selection as both factors may impact one another in relation to adolescent friendship networks.

Past studies also demonstrate that parental influences affect adolescent drinking behavior directly. Previous research indicates that parental support prevents the early initiation of alcohol use and reduces the frequency of alcohol use among adolescents [40,41,42]. Moreover, parental monitoring and supervision have been negatively associated with adolescent alcohol use [19,43]. Not all parental influences, however, are protective for alcohol use, as youth in homes in which parents drink or parents display high levels of permissiveness for adolescent drinking are more likely to increase their alcohol use levels over time [44,45,46].

It is possible that peer and parental influences may function together in impacting adolescent friendship tie choices and drinking behavior, given insights from ecological models of development suggesting that influences from these two contexts may act synergistically [10]. In a study of 4,230 7th to 12th graders, parental drug (including binge drinking) attitudes had an indirect effect on the risk of adolescent drug use, which was mediated through peer drug use [47]. In another study, Marshal and Chassin [48] found that parental support and discipline buffered the effects of peer group affiliation on alcohol use of female adolescents. It may be that parental attitudes and behavior relevant to monitoring, support, and parental drinking operate synergistically with youths' affiliations with peers who use alcohol in affecting youths' friendship tie choices. Having said that, extant studies have not systematically examined these parental influences as they affect the co-evolving processes of friendship tie choice behavior and alcohol use among adolescents, nor have they examined the interactions under study. As such, whereas past studies have found a positive peer influence effect on adolescent alcohol use [15–20], the current study goes a step further to examine whether youth whose parents engage in close monitoring or provide strong emotional support, or have a home environment condoning drinking behavior, are more or less likely to adopt the drinking behavior of their friends through a peer influence effect on their own drinking behavior. Secondly, the current study also assesses whether parental support, parental monitoring, and the parental home drinking environment interact with whether youth choose friends who drink as both factors might multiplicatively affect youths' friendship tie choice.

Finally, we also examine the effect of depressive symptoms among adolescents on their alcohol use. Adolescent drinking has been positively related to depression in previous studies [23,49–53]. Depressive symptoms in adolescent youth have also been related to youths' friendship tie choice behavior [54]. As such, we examine the role of depressive symptoms as they relate to adolescent drinking behavior.

The current study builds upon extant literature examining the importance of peer influence and selection processes related to adolescent friendship tie choice and alcohol use. In addition, we also focus on the role of key parental influences on adolescent friendship tie choice and alcohol use behavior. With data from the AddHealth study, we explore the co-evolution of adolescents' friendship ties and their drinking behavior over two waves. We hypothesize that we will observe both peer influence and peer selection effects, as adolescents will select friends with similar alcohol use levels, and adjust their drinking behavior based on the drinking behavior of their friends. We also expect that parental monitoring, parental support, and the parental home drinking environment, will influence the friendship tie choice and adolescent drinking, both directly and synergistically.

## Data and Methods

### Data

The data utilized in this study come from early waves of AddHealth. The respondent record/information was anonymized and de-identified prior to analysis. This study has been approved by the Institutional Review Board of the University of California, Irvine (2013). This study does not employ human subjects directly, as our analyses utilize secondary data which are de-identified. AddHealth participants provided written informed consent for participation in all aspects of AddHealth in accordance with the University of North Carolina School of Public Health Institutional Review Board guidelines that are based on the Code of Federal Regulations on the Protection of Human Subjects 45CFR46: http://www.hhs.gov/ohrp/humansubjects/guidance/45cfr46.html. Written informed consent was given by participants (or next of kin/caregiver) for their answers to be used in this study.

AddHealth is a longitudinal study of a stratified sample of US schools from 7th to 12th grades [55]. The AddHealth data is one of the richest adolescent network data sources collected to date in the United States. The network boundary is defined by a meaningful social and policy-relevant unit, the school, with information on basic demographics (i.e., gender, race, age), attitudes and behaviors, and ecological structures (i.e., family, school, neighborhood), as well as on all friendship relations. Thus these data are ideal for examining the co-evolution of friendship networks and drinking behavior in school based contexts. Although the AddHealth data were collected nearly 20 years ago, these data continue to be relevant to the mechanisms of in-school friendship formation, and continue to be widely used in current public health studies [32,56–62].

AddHealth contains a saturated sample of 16 schools out of the total 132 participant schools [55]. Among the 16 schools, there is a special education school with constant student turnover, and another school with an administrative error in which the students' IDs at the earlier wave could not be matched with those at later waves. Thus these two schools are not suitable for longitudinal network analysis. Of the remaining 14 schools, two are large schools (over 1,000 students enrolled) often called "Jefferson High" and "Sunshine High" [56,63] whose macro settings were quite different from the other 12 small schools with fewer than 200 students enrolled [64,65]. (Among the twelve small schools, network size, or number of respondents, is between 30 and 197. The mean is 107 and SD is 53.46.) Given computational power issues, we could not estimate the model for the biggest school, "Sunshine High." Since our analysis requires longitudinal measures of friendship networks, we focus on two samples: 1) a saturated sampling of 1,284 students from 12 small schools, and 2) that of 976 students from the second biggest school, "Jefferson High" [63].

During the administration of the AddHealth survey, all students in 14 schools were invited to take the survey over three waves. Information on the social and demographic characteristics of the respondents was collected, as well as their risk behaviors including alcohol use. Adolescent sociometric networks were constructed from a network elicitation item asking respondents to name up to five male and five female best friends from a name list of students in his/her school, and thus researchers are able to attain longitudinal complete sociometric networks in these schools. The measures of youths' parental contexts came from a parent survey between April and December of 1995, the same time when the respondents also took a wave 1 In-Home Survey. However, due to another administrative error, about 37% respondents in the 12 small schools and about 5% respondents in "Jefferson High" were recorded to nominate only one (instead of five) female and male best friends. To overcome this limited nomination difficulty, in this study we utilize the information retrieved from the first (In-School Survey during 1994 and 1995) and third (wave 2 In-Home Survey between April and December of 1996) time points for our dynamic network analysis, skipping that from the second time point (wave 1 In-Home Survey).

### Measures

The dependent (behavior) variable measures drinking frequency in the past 12 months. At wave 1, the survey question was "During the past twelve months, how often did you drink beer, wine, or liquor?" with response categories of "0—never", "1–1 once or twice", "2–2 once a month or less", "3–2 or 3 days a month", "4—once or twice a week", "5–3 to 5 days a week", and "6—nearly every day." In waves 2 and 3, the survey question was "During the past 12 months, on how many days did you drink alcohol?" with response categories of "1—every day or almost every day", "2–3 to 5 days a week", "3–1 or 2 days a week", "4–2 or 3 days a month", "5—once a month or less (3–12 times in the past 12 months)", "6–1 or 2 days in the past 12 months", and "7—never". We recoded these such that response categories specify non-drinkers (0 = never), casual-drinkers (1 = 1 or 2 days), light-drinkers (2 = once a month or less or 3–12 times in the past 12 months), medium-drinkers (3 = 2 or 3 days a month), and heavy-drinkers (4 = more than 1 or 2 days a week). (We do not have a measure of drinking intensity. The only related question asked over all three time points was "During the past twelve months, how often did you get drunk?" However, students may interpret the number of drinks required to be "drunk" very differently, and we therefore do not include this measure.)

Predictors of drinking behavior include *gender* (0 = male, 1 = female), *grade* (7~12), depressive symptoms, parental support, parental monitoring, and the parental home drinking environment. *Depressive symptom status* is generated as a factor score of 19 ordinal items modified from the Center for Epidemiologic Studies Depression Scale (CES-D; Cronbach's α = 0.87) [66]. *Parental support* and *parental monitoring* are computed as standardized factor scores (means = 0, standard deviations = 1) through confirmatory factor analysis, with Root Mean Squared Error of Approximation (RMSEA) about. 05 and Comparative Fit Index (CFI) greater than. 95, which both suggest a good fit. Items indicating *parental support* include whether the student had talked about a personal problem with their parents (0 = no, 1 = yes), whether the parents and the student communicated well, whether the parents were warm and loving, whether the student reported a "good relationship" with parents (same response categories for three items above: 1 = strongly disagree, 2 = disagree, 3 = neither agree nor disagree, 4 = agree, 5 = strongly agree), the student's closeness to their parents, and how much the student felt his or her parents cared about him or her (same response categories for two items above: 1 = not at all, 2 = very little, 3 = somewhat, 4 = quite a bit, 5 = very much). Items indicating *parental monitoring* include whether parents let the student make decisions about a weekend curfew, the people the student hung around with, how much television the student watched, which television program the student watched, and what the week night bedtime was (0 = yes, 1 = no), and the presence of parents when the student was back from school (0 = never, 1 = almost never, 2 = some of the time, 3 = most of the time, 4 = always, 5 = they brought the student home from school), eating dinner (0~7 days per week), and going to bed (0 = never, 1 = almost never, 2 = some of the time, 3 = most of the time, 4 = always). Home drinking environment is measured by summing up two binary measures (0 = no, 1 = yes): parental drinking was coded as "yes" if the parent reported that they drank at least once a month, and alcohol availability was coded as "yes" if the adolescent respondents reported positively that "alcohol is easily available at home".

### Plan of Analysis

To explore the co-evolution of friendship networks and drinking behavior in continuous Markov time, we utilize the SAB model with the R-based Simulation Investigation for Empirical Network Analysis (RSiena) package [67]. The SAB model assumes that a respondent will make decisions that optimize his or her network and behavior status in the next time step based on his or her current state of network-behavioral configuration, which is referred to as the objective function. The objective function is defined as f(β,x) = Σ_k_β_k_S_ik_(x), where β_k_ is the *k*th estimated parameter for the actor-specific effect s_ik_(x) and x is the joint network-behavioral state. Positive values of the objective function indicate the preferred direction of changes, while negative values suggest the avoidance of such changes. In RSiena, the objective function of network changes and behavior changes are estimated simultaneously to generate both a network and a behavioral equation. Together, these constitute a set of interdependent equations with the rate functions λ_i_(α,x), which indicate the expected frequency of changes in the networks or behavior the actors make between observation points. The model is then estimated by simulating the networks and behavior forward in time. Thus, there are many micro-steps in the model in which actors update their objective functions regarding alcohol use behavior and network tie choice. A Method of moments (MoM) estimation is used to estimate the network and behavior parameters such that the main characteristics of the networks and behaviors are most closely approximated.

As shown in Table 1, in the network equation predicting friendship tie choice, we include several structural network effects, i.e., out-degree and reciprocity capturing tie preference, transitive triplets and three cycles measuring triadic closure, and in-degree popularity and in-in degree assortativity (square root) differentiating the tendency towards preferential attachment vs. degree assortativity [68,69]. The network equation (friendship tie choice) also controls for similarity measures, including similarity on gender, grade, and parent education level (as a proxy of family socio-economic status). The function of parental influences on friendship tie choice is tested by the inclusion of respondents' parental support, parental monitoring, and their parental home drinking environment. For the behavior variable alcohol use (*z*), we specify it as a main effect on alter attractiveness (drinking alter), as a main effect on network activity of ego (drinking ego), and as a similarity (homophily) effect.

**Table 1:** Effects for modeling network evolution (friendship tie choice).

Effect	Description
Friendship tie choice rate parameter	The expected number of change opportunities for each ego during each period
Out-degree (density)	Propensity to nominate a friend
Reciprocity	Propensity to have mutual friendships
Transitive triplets	Propensity to become the friend of a friend
Three cycles	Propensity to choose a friendship nominator's nominator as a friend
In-degree popularity	Propensity to choose a popular youth for a friend
In-in degree assortativity (square root)	Propensity to choose an adolescent similar in in-degree as a friend
Drinking alter (friend)	Effect of friends' drinking behavior on friendship tie choice
Respondent (ego) covariates: drinking, parental variables	Effect of respondent's or parental behavior on friendship tie choice
Drinking similarity, gender similarity, grade similarity, parental education similarity	Propensity to have ties to similar adolescents (selection effect)
Moderating effect	Propensity for those with higher values of covariate to choose friends who drink (+) or the tendency for an adolescent with higher values of covariate to choose friends who drink less (-)

As shown in Table 2, in the drinking behavior equation, the linear and quadratic shape parameters model the long-term trend in alcohol use. The in-degree item measures whether adolescents receiving more in-coming ties (more popular) drank more over time. We measure the peer influence effect as the sum of negative absolute difference between ego's and alters' behavior averaged by ego's out-degree. Additional covariates such as gender, grade, and depressive symptoms are controlled to test how the parental factors—i.e., parental home drinking environment, parental support, and parental monitoring—affected adolescent drinking in Model 1.

**Table 2:** Effects for modeling behavioral evolution (alcohol use).

Effect	Description
Drinking rate parameter	The expected number of change opportunities for each ego in each period
Linear shape	The basic drive toward high values of drinking
Quadratic shape	The self-reinforcing function of drinking behavior
In-degree	Propensity for popular student to have high values of drinking
Peer influence	Effect of drinking behavior similarity between respondent and each alter (Peer Influence effect)
Covariate: parental variables, gender, depressive symptom	Effect of covariate on drinking
Moderating effect	Propensity for an adolescent with a higher value of a covariate to have a higher propensity to match alters' behavior

Whereas Model 1 includes all the measures described (the "main effects" model), Models 2 through 4 assess the moderating role of an adolescent's parental factors. Thus, we included interaction variables of selecting a friend who drank (drinking alter) with parental support, parental monitoring, and the parental home drinking environment in Models 2, 3, and 4, respectively. Likewise, in these same models we included interaction terms of friends' influence effect (peer influence) with parental support, parental monitoring, and the parental home drinking environment in Models 2, 3, and 4, respectively.

It should be noted that when the interaction between parental support and friends' influence was added to the behavior equation of Model 2 for the 12 small schools, many of the effects were dramatically inconsistent with that in Model 1, 3, and 4: both their parameters and standard errors appear to be magnified (as shown in the shaded area of S1 Table). A further examination of the correlations among the parameters suggests collinearity issues due to this specific interaction effect. We therefore removed this interaction from the behavior equation of Model 2 for the 12 small schools. We have also tried estimating a Model 5 for each sample, with all potential moderators included in the same SAB model. However, due to the high collinearity among these interaction effects, the model cannot reach convergence.

There are various approaches to combining multiple networks, including a meta-analysis approach and combining different sub-projects into one multi-group project with rate parameters allowed to differ across sub-projects. In this study we followed Cheadle and Goosby [64] and Cheadle and Schwadel [65] to explore the general tendency of the network and behavior dynamics in the 12 small schools by combining their friendship networks into one large network and using structural zeroes to indicate that ties between the schools are not permitted (see [67], page 81).

Although we aimed to explore the general tendency of network and behavior dynamics in the 12 small schools, we also considered possible variations across schools along several key dimensions: (1) urban, suburban vs. rural, (2) public vs. private, (3) single race vs. multiple race, and (4) different response rates. For the first three dimensions, we estimated ancillary models including interactions between dummy variables for these contextual variables and key drinking effects. They were insignificant, suggesting that co-evolution of friendship tie choice and drinking behavior was similar across different types of schools (e.g., see S2 Table along with S1 File). Among the 12 small schools, 10 had response rates above 70% and 2 had response rates between 55%~70%. To account for the influence from response rate, we ran ancillary models with friendship networks of the 10 small schools combined into one large network and compared the results with those from the 12 small schools. The parameters were quite similar in these separate models. These results are available from the authors upon request.

In this way we can list the results of the 12 small schools and "Jefferson High" side by side to observe their similarities and differences. Respondents showing up at either an early or later observation point are included in the analysis. They were also allowed to join or leave their networks (e.g., graduates, movers, dropouts), with structural zeroes indicating they were no longer there at this time point (see [67,70]). Missing data are handled by RSiena software and imputed within the models as Huisman and Steglich [71], and Ripley, Snijders, Boda, Vörös, and Preciado [67] suggested. We assessed the goodness-of-fit of the models by comparing network statistics and drinking distribution of 1,000 simulations based on our model to the observed network and the fit was quite good (e.g., see S1 and S2 Figs. along with S2 File).

## Results

### Descriptive Results

The alcohol use and network descriptive statistics of the 12 small schools and "Jefferson High" are summarized in Table 3. (The distribution of drinking behavior in each of 12 small schools is shown in S3 Table.) Among the small school sample, 52% students reported they were non-drinkers during the In-School Survey, and this proportion increased to 61.1% during the wave 2 In-Home Survey. Although the number of non-drinkers also increased in "Jefferson High", as suggested by the proportion of light- (2 = once a month or less or 3–12 times in the past 12 months), medium- (3 = 2 or 3 days a month), and heavy-drinkers (4 = more than 1 or 2 days a week), drinking was a far more prevalent behavior in this school than in small schools. The group size of casual-drinkers (1 = 1 or 2 days) decreased in both samples, and their members either became non-drinkers, or chose to increase their drinking frequency to other levels.

**Table 3:** Behavior and network descriptive statistics.

	12 small schools (*n* = 1,284)		Jefferson High (*n* = 976)	
	In-School Survey	wave 2 In-Home Survey	In-School Survey	wave 2 In-Home Survey
Alcohol use (past 12 months, %)				
0 = never	52.02	61.06	30.53	37.60
1 = 1 or 2 days	22.12	13.32	23.46	13.73
2 = once a month or less (3–12 times in the past 12 months)	7.94	10.12	12.70	15.98
3 = 2 or 3 days a month	5.84	6.23	13.63	14.04
4 = more than 1 or 2 days a week	12.07	9.27	19.67	18.65
Network statistics				
Out-going ties	6,671	2,704	6,063	2,484
Reciprocal index	0.45	0.33	0.34	0.35
Transitive index	0.34	0.34	0.18	0.20
Jaccard index	0.22		0.21	

*Note*: The reciprocity index indicates the proportion of ties that were mutual. The transitivity index is the proportion of 2-paths (ties existing between AB and BC) that were transitive (ties existing between AB, BC, and AC). The Jaccard index measures the network stability between successive waves.

Among the 1,284 respondents in the 12 small schools, the number of out-going ties decreased from 6,671 during the In-School Survey to 2,704 during the wave 2 In-Home Survey due to graduation, moving, dropping out, and attrition/non-response/missing network data. A similar pattern was also observed in "Jefferson High". The proportional change of reciprocal ties was more variable in the small schools (from 0.45 to 0.33). The transitivity index, which captures the tendency for individuals to experience triadic closure, was found to be relatively stable over time, although stronger in small schools (34%) compared to "Jefferson High" (18~20%). As indicated by the Jaccard index, there was a high turnover of friendship ties in both samples, with only 21~22% of ties persisting over the two waves.

The descriptive statistics of covariates are reported in Table 4.

**Table 4:** Descriptive statistics of covariates.

		12 small schools (*n* = 1,284)	Jefferson High (*n* = 976)
In-School Survey	Female (%)	50.93	48.46
	Grade level (%)		
	7th grade	23.99	0.00
	8th grade	24.92	0.00
	9th grade	14.25	28.79
	10th grade	12.69	28.38
	11th grade	12.00	21.82
	12th grade	12.15	21.00
	Parent education level (%)		
	Less than high school	6.70	5.02
	High school	38.86	38.22
	Some college or trade school	30.84	37.09
	Graduate of college/university	23.60	19.67
	Depressive symptoms, mean (SD)	-0.12 (0.46)	0.01(0.53)
Parent Survey	Parental support, mean (SD)	0.06 (0.25)	-0.05(0.29)
	Parental monitoring, mean (SD)	0.02 (0.12)	-0.04(0.10)
	Parental home drinking environment, mean (SD)	0.87 (0.78)	1.19(0.73)

### Network Evolution: Friendship Tie Choice

As shown in the network equation of Model 1 in Table 5 and Table 6, we observe significantly positive parameters for drinking similarity in both samples, although the estimated parameter is about 135% larger in the 12 small schools (*b* = .33, *p* <. 01) than in "Jefferson High" (*b* = .14, *p* <. 05). These provide evidence of a peer selection effect, as students were more likely to select as friends others with similar levels of alcohol use.

**Table 5:** Stochastic Actor-Based model of friendship tie choice and adolescent drinking behavior for 12 small schools (*n* = 1,284).

Effect name	Model 1		Model 2a		Model 3		Model 4	
Network decision: Friendship tie choice	beta	s.e.	beta	s.e.	beta	s.e.	beta	s.e.
Constant friendship rate (period 1)	15.72***	0.54	15.62***	0.63	15.57***	0.69	15.56***	0.76
Out-degree (density)	-2.09***	0.32	-2.03***	0.15	-2.01***	0.16	-1.89***	0.24
Reciprocity	1.79***	0.15	1.79***	0.09	1.78***	0.10	1.79***	0.09
Transitive triplets	0.23***	0.03	0.23***	0.03	0.23***	0.03	0.22***	0.03
3-cycles	-0.14**	0.05	-0.14*	0.06	-0.14*	0.05	-0.14*	0.06
In-degree popularity	0.07***	0.01	0.07***	0.01	0.07***	0.01	0.07***	0.01
In-in degree^(1/2) assortativity	-0.07**	0.02	-0.07*	0.03	-0.07*	0.03	-0.07*	0.03
Gender similarity	0.20***	0.03	0.20***	0.03	0.20***	0.04	0.20***	0.04
Parental education similarity	0.03	0.03	0.04	0.03	0.04	0.03	0.04	0.02
Grade similarity	0.45***	0.03	0.45***	0.03	0.45***	0.03	0.45***	0.02
Parental support ego	0.35***	0.09	0.58**	0.19	0.35***	0.11	0.37***	0.10
Parental monitoring ego	0.07	0.20	0.02	0.25	-0.42	0.29	0.06	0.20
Parental home drinking environment ego	-0.03	0.05	-0.04	0.03	-0.04	0.03	-0.23***	0.06
Drinking alter	0.28*	0.12	0.28**	0.10	0.29***	0.07	0.09†	0.05
Drinking ego	-0.07	0.11	-0.06	0.09	-0.06	0.06	-0.05	0.07
Drinking similarity	0.33**	0.11	0.31***	0.07	0.35***	0.06	0.29***	0.09
Parental support ego x Drinking alter			-0.20	0.13				
Parental monitoring ego x Drinking alter					0.30	0.31		
Parental home drinking environment ego x Drinking alter							0.18*	0.06
Behavior decision: Alcohol use								
Rate drinking behavior (period 1)	24.03***	2.46	23.89***	3.12	21.10***	1.29	23.22***	2.29
Drinking behavior linear shape	-1.78***	0.13	-1.73***	0.18	-1.75***	0.23	-1.82***	0.30
Drinking behavior quadratic shape	0.30***	0.02	0.30***	0.01	0.29***	0.02	0.30***	0.02
Drinking behavior in-degree	0.01	0.01	0.01	0.01	0.02†	0.01	0.01†	0.01
Drinking behavior peer influence	0.22*	0.10	0.23*	0.10	0.32*	0.15	0.10	0.17
Effect from gender (female = 1)	-0.06	0.05	-0.06	0.04	-0.06	0.05	-0.06	0.04
Effect from grade	0.03*	0.01	0.04*	0.02	0.03†	0.02	0.03†	0.02
Effect from depressive symptoms	0.00	0.05	0.00	0.04	0.00	0.05	0.00	0.05
Effect from parental home drinking environment	0.10***	0.03	0.10***	0.02	0.11***	0.03	0.09*	0.04
Effect from parental support	-0.01	0.07	0.02	0.10	-0.01	0.09	-0.01	0.11
Effect from parental monitoring	-0.39*	0.18	-0.43*	0.19	-0.10	0.26	-0.39*	0.19
Effect from parental monitoring x peer influence					3.08†	1.84		
Effect from parental home drinking environment x peer influence							-0.11	0.17

† Two-sided p<0.1.

* Two-sided p<0.05.

** Two-sided p<0.01.

*** Two-sided p<0.001.

**Table 6:** Stochastic Actor-Based model of friendship tie choice and adolescent drinking behavior for "Jefferson High" (*n* = 976).

Effect name	Model 1		Model 2		Model 3		Model 4	
Network decision: Friendship tie choice	beta	s.e.	beta	s.e.	beta	s.e.	beta	s.e.
Constant friendship rate (period 1)	35.55***	2.51	35.01***	2.19	35.23***	2.09	35.42***	1.85
Out-degree (density)	-2.51***	0.24	-2.47***	0.26	-2.37***	0.22	-2.46***	0.29
Reciprocity	2.73***	0.11	2.75***	0.10	2.73***	0.11	2.74***	0.12
Transitive triplets	0.51***	0.04	0.51***	0.04	0.51***	0.04	0.51***	0.04
3-cycles	-0.43***	0.07	-0.43***	0.09	-0.44***	0.09	-0.42***	0.08
In-degree popularity	0.04*	0.02	0.04*	0.02	0.03***	0.01	0.04**	0.01
In-in degree^(1/2) assortativity	-0.03	0.03	-0.04	0.03	-0.03	0.02	-0.04	0.04
Gender similarity	0.27***	0.04	0.27***	0.06	0.27***	0.04	0.27***	0.06
Grade similarity	0.41***	0.03	0.41***	0.03	0.41***	0.02	0.41***	0.03
Parental education similarity	0.04	0.02	0.04	0.02	0.04	0.03	0.04	0.03
Parental support ego	0.03	0.08	0.31	0.68	0.03	0.08	0.04	0.07
Parental monitoring ego	-0.31	0.23	-0.30	0.24	-0.76	0.88	-0.30	0.27
Parental home drinking environment ego	0.00	0.04	0.00	0.02	-0.01	0.03	-0.06	0.08
Drinking alter	0.11**	0.04	0.10	0.10	0.12**	0.04	0.07	0.09
Drinking ego	-0.08*	0.04	-0.07*	0.04	-0.08**	0.03	-0.08†	0.04
Drinking similarity	0.14*	0.06	0.13*	0.05	0.14***	0.04	0.13*	0.04
Parental support ego x Drinking alter			-0.14	0.28				
Parental monitoring ego x Drinking alter					0.25	0.52		
Parental home drinking environment ego x Drinking alter							0.03	0.05
Behavior decision: Alcohol use								
Rate drinking behavior (period 1)	21.82***	2.06	21.61***	2.17	21.17***	2.61	21.04***	2.73
Drinking behavior linear shape	-1.36***	0.25	-1.57***	0.40	-1.27***	0.22	-1.40***	0.24
Drinking behavior quadratic shape	0.23***	0.03	0.23***	0.02	0.23***	0.02	0.23***	0.03
Drinking behavior in-degree	0.01†	0.01	0.01	0.01	0.01	0.01	0.01	0.01
Drinking behavior peer influence	0.36*	0.16	0.29†	0.15	0.32*	0.14	-0.09	0.71
Effect from gender (female = 1)	-0.09**	0.03	-0.10**	0.04	-0.10*	0.04	-0.10*	0.05
Effect from grade	0.02	0.03	0.04	0.03	0.01	0.02	0.03	0.03
Effect from depressive symptoms	0.03	0.07	0.03	0.04	0.03	0.06	0.03	0.05
Effect from parental home drinking environment	0.06*	0.03	0.06**	0.02	0.06*	0.03	0.05	0.04
Effect from parental support	-0.07	0.12	-0.01	0.18	-0.08	0.08	-0.07	0.08
Effect from parental monitoring	-0.34	0.30	-0.28	0.17	-0.49†	0.29	-0.35	0.29
Effect from parental support x peer influence			-0.31	6.98				
Effect from parental monitoring x peer influence					-1.75	2.97		
Effect from parental home drinking environment x peer influence							0.38	0.52

† Two-sided p<0.1.

* Two-sided p<0.05.

** Two-sided p<0.01.

*** Two-sided p<0.001.

In both samples we also find that those drinking more frequently were more popular than those drinking less frequently, although they seemed to be even appealing in small schools (*b* = .28, *p* <. 05) compared to "Jefferson High" (*b* = .11, *p* <. 01). Adolescents having higher drinking levels were inclined to nominate fewer best friends that those with lower drinking levels, but this effect is only significant in "Jefferson High" (*b* = -.08, *p* <. 05).

In terms of structural network effects, the significantly negative out-degree (*b* = -2.09, *p* <. 001 in small schools and *b* = -2.51, *p* <. 001 in "Jefferson High") and the significantly positive reciprocity parameters (*b* = 1.79, *p* <. 001 in small schools and *b* = 2.73, *p* <. 001 in "Jefferson High") suggest that adolescents in both samples tended to form fewer ties, but when they did such ties were more likely to be reciprocal ones. The adolescents also preferred to be friends with their friends' friends (triadic closure), as indicated by the positive transitive triplets effect (*b* = .23, *p* <. 001 in small schools and *b* = .51, *p* <. 001 in "Jefferson High"). The significantly negative three-cycle effect implies a tendency toward local hierarchy (*b* = -.14, *p* <. 01 in small schools and *b* = -.43, *p* <. 001 in "Jefferson High"). Adolescents were more likely to be named as a tie if they already had many in-coming ties (high in-degree popularity) (*b* = .07, *p* <. 001 in small schools and *b* = .04, *p* <. 05 in "Jefferson High"). And the negative parameter for in-in degree assortativity (square root) again suggests the presence of preferential attachment as adolescents with high in-degree were more likely to be nominated as best friends by those with low in-degree, but this effect is only significant in the small school sample (*b* = -.07, *p* <. 01).

We find that homophily preferences in gender and grade drove friendship tie formation: adolescents were more likely to send friendship nominations to other adolescents with the same gender (*b* = .20, *p* <. 001 in small schools and *b* = .27, *p* <. 001 in "Jefferson High") and in the same grade (*b* = .45, *p* <. 001 in small schools and *b* = .41, *p* <. 001 in "Jefferson High"). The homophily effects of parental educational levels are insignificant in both samples, after controlling for the other measures in the model.

As for how the parental influences affected friendship tie choice, we find that adolescents who received more parental support nominate more friends in the 12 small schools (*b* = .35, *p* <. 001). This effect for parental support was insignificant in "Jefferson High". And we find that more parental monitoring and a home drinking environment did not affect tie choice.

**Fig 1:**
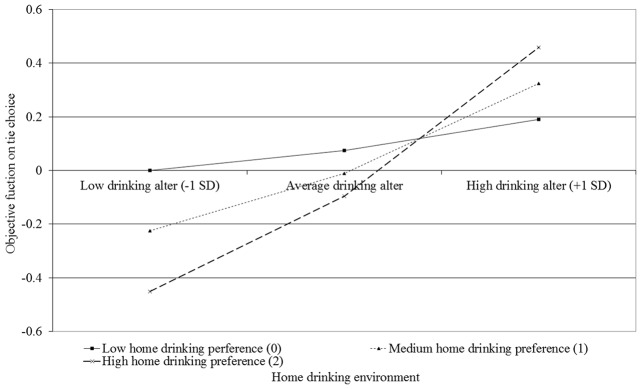
Interaction of home drinking environment ego and drinking alter on tie choice in Model 4 for the 12 small schools.

When focusing on the moderating effect of family contexts on friendship tie choice in Models 2 to 4 of Tables 5 and 6, although the signs of interaction parameters are as expected, the only statistically significant effect is that between the parental home drinking environment and drinking alter in the small school sample (*b* = .18, *p* <. 05): whereas higher-level drinkers were more popular in general, those with high levels of drinking in their home environment were particularly likely to form ties with those who drink more, see Fig. 1.

### Behavior Evolution: Alcohol use

As shown in the behavior equation of Model 1 in Table 5 and Table 6, we find a significant peer influence effect (*b* = .22, *p* <. 05 in the small schools and *b* = .36, *p* <. 05 in "Jefferson High"), implying that adolescents tended to match the drinking behavior of their friends (i.e., as the drinking frequencies of their friends increased, so did their drinking propensity over time).

The significantly negative linear shape effect (*b* = -1.78, *p* <. 001 in small schools and *b* = -1.36, *p* <. 001 in "Jefferson High") and the significantly positive quadratic shape effect (*b* = .30, *p* <. 001 in small schools and *b* = .23, *p* <. 001 in "Jefferson High") suggest that adolescents tended to drink less over time, but there was also a self-reinforcement effect of alcohol use: there appeared to be a tendency towards polarization on both ends of drinking behavior, as adolescents were inclined to either become or remain a non-drinker or escalate to heavy use. Popular adolescents (with higher values of in-degree) were not found to drink more frequently in either case. Additional significant effects were found for grade in small schools (*b* = .03, *p* <. 05) and gender in "Jefferson High" (*b* = -.09, *p* <. 01): higher-grade adolescents in the 12 small schools and males in "Jefferson High" were generally found to increase their drinking frequency over time more than their counterparts.

Regarding the parental influences, we find that adolescents who experienced more drinking at home were more likely to increase their own drinking frequency (*b* = .10, *p* <. 001 in small schools and *b* = .06, *p* <. 05 in "Jefferson High"). Adolescents with higher levels of parental monitoring engaged in less drinking behavior over time, but this effect is only significant in small school sample (*b* = -.39, *p* <. 05). The parental support respondents received had a negative effect on the frequency of adolescent drinking, although not significant in either case.

Turning to the interaction effects included in Model 2 to Model 4 of Table 5 and Table 6, there was no evidence that parental support, parental monitoring, or the parental home drinking environment moderated the peer influence effect in either sample.

## Discussion

This study aimed to disentangle peer selection and peer influence processes and other key network effects simultaneously with salient parental influences by examining the co-evolution of friendship tie choices and drinking behavior among adolescents in two AddHealth samples. Because the network size and drinking prevalence were relatively different in the two samples—e.g., Jefferson High was a much larger school with intensive adolescent alcohol use—one might presume that the co-evolution mechanisms of friendship tie choices and drinking behavior would differ. Our models demonstrate that to the contrary, the evidence across the two samples was typically quite similar. In the friendship tie choice equation, most measures had effects similar in direction and statistical significance. The differences were mostly in magnitude of the effects: in the friendship tie choice equation, whereas the network structural effects such as reciprocity, transitive triplets, and three-cycles were larger in "Jefferson High", the popularity of heavier drinkers and the selection effect of choosing ties based on similarity of drinking behavior were smaller in "Jefferson High". The effect of peer influence was stronger in "Jefferson High" with more drinking behavior, implying that such a context may be particularly important for transmitting norms about drinking behavior among adolescents. The most notable differences were that in small schools with lower levels of alcohol use, parental support had a much stronger effect on friendship tie choice, the parental home drinking environment had a much stronger effect on forming ties with those who drink more, and parental monitoring appeared more effective in reducing drinking behavior.

Our findings demonstrate that peer selection played a significant role in facilitating drinking behavior similarity in the adolescents' friendship networks. Adolescents preferred to form friendships with those who displayed similar levels of alcohol use. These results support previous findings regarding the importance of peer selection in accounting for behavioral similarities across dyads in friendship networks [32,34]. At the same time, our findings also indicate the important role of peer influence in friendship networks, as do previous studies [33,34], demonstrating the importance of peer influence in adolescent networks. Overall, our findings suggest that both peer and parental factors were instrumental in shaping both friendship tie choice and drinking behavior.

Regarding our findings pertaining to friendship tie choice, we observe that heavy-drinkers received more in-coming ties and hence were more popular. Also using data from the AddHealth In-School survey, Balsa, Homer, French, and Norton [21] found that popularity (also measured as ego's in-degree) was positively associated with drinking frequency. However, they acknowledged that their cross-sectional design prevented them from assessing the causal ordering or the extent that a reciprocated relationship existed between these two measures [21]. Our findings suggest a unilateral relationship between alcohol use and network popularity: whereas alcohol use increased popularity, more popular adolescents did not drink more over time.

Our findings also suggest that while adolescents' peer relationships were central to their lives, parents still had influence on both adolescent alcohol use and friendship tie choice. First, in line with previous studies [44,45,46], adolescents were more likely to engage in underage drinking if the parents provided a favorable environment for adolescent alcohol use. In our study, these respondents in such family environments were not only particularly likely to form friendship ties with adolescents with high drinking levels, but our model implies that through peer influence they would over time engage in higher levels of alcohol use.

We also observed a negative relationship between parental monitoring and drinking in the small school sample. Our finding is consistent with prior studies indicating a negative relationship between parental monitoring and adolescent drinking behavior [19,43]. That parental monitoring is risk protective for substance use has been shown in these studies, which highlight the continuing role of parents in enforcing rules and discipline during the high school years helped reduce the alcohol use level among adolescents, at least in the small school sample.

Our findings also indicated a negative relationship between parental support and alcohol use. This finding is consistent with past studies indicating a negative relationship between parental support and drinking behavior [40,41,42]. It is likely that the provision of parental support renders adolescents more able to develop social competencies necessary to form friendship ties.

Our findings differ from a previous study using Add Health [32] that did not find a significant influence effect. There are several reasons for these different results. First, there is different sample selection, as our study utilizes data from the In-School Survey and the wave 2 In-Home survey, but not data from the wave 1 In-Home Survey to avoid difficulty due to limited nominations, whereas Mundt and colleagues [32] selected respondents from the wave 1 In-Home survey and the wave 2 In-Home Survey. Second, our study includes respondents present in either wave, whereas Mundt and colleagues [32] only included those who completed the survey at the first time point (assuming those not completing the survey are not part of the network). Third, our study integrates parental factors into the friendship tie choice and drinking equations, which had important effects in the models. Fourth, we utilized a different strategy for handling multiple networks, as we combined the friendship networks of the 12 small schools into one large network, whereas Mundt and colleagues [32] employed a meta-analysis. Finally, we utilized a different peer influence specification. Each of these factors likely contributes to the differences in results across these two studies.

Our study has several limitations. First, all the analyses are based on self-reported data from AddHealth. One consequence of using self-reports of illicit substance use is underreporting. Some studies find that adolescent reporting is better than parent, peer, or other reporting [72]. Still, future studies may benefit by using multiple measures of alcohol use (e.g. employing physiological or biological indicators of alcohol use) to ensure high internal validity [73]. Second, the friendship networks were retrieved through a name generator limited to a maximum of five male and five female best friends and thus were not a fully accurate portrayal of a respondent's peer network. It is unclear how our findings would have differed had the adolescents sampled been allowed to nominate *all* of their friends. Third, the AddHealth data did not include information on alcohol use behavior of other family members, i.e., respondents' brothers and sisters. It would be much better for future studies to account for various levels of familial influences.

Despite these limitations, our findings have implications for future studies. Our findings suggest merit in further examination of the role of the parental influences under study as they affect co-evolution of friendship networks and drinking behavior among US adolescents, as well as the mechanisms underlying the relationship between alcohol use and network popularity. Given that those in a home environment which favored drinking were particularly likely to form friendship ties with higher-drinking-level adolescents suggests a need to study this possibility more closely.

Our findings also have practical implications for health behavior change interventions targeting adolescent alcohol use. Motivated by intuition from intervention studies applying concepts from the opinion leader literature [74,75] employing the general strategy of identifying popular youth as means to transmit prosocial peer influences through a network system, we suggest that one way to promote positive peer influences against drinking and to likewise dampen the influence of drinkers in peer networks is to target popular youth who do not drink and are connected through transitive triplets. Given the hierarchical structuring of our data (as indicated by the significantly positive transitive triplet and significantly negative three cycle effect), popular youth are uniquely positioned, and as well uniquely salient in the highest rank of the hierarchy. Peer influences exerted by popular youth will likely diffuse down the social hierarchy to less popular youth. In addition, given the salience of reciprocated ties among adolescent youth, another intervention strategy would target mutually reciprocated friendship pairs of youth (i.e., drinking pairs, non-drinking pairs, and mixed drinking status pairs) to promote anti-drinking peer influences, social support, and resistance training skills to influence one another to stop drinking or not begin drinking. Lastly, parents should be targeted to both increase their capacity to provide support to and monitoring of their adolescent children, in order to help their children foster friendships with prosocial peers who are not substance users.

## Conclusion

In sum, this study examined the co-evolution of friendship networks and drinking behavior among two representative samples of US adolescents. Adolescents with similar alcohol use levels were more likely to form friendships than their peers with more dissimilar alcohol use levels, and adolescents also adjusted their drinking behavior to match that of their best friends. Moreover, we found that those who drank more were more popular, but popular adolescents did not drink more over time. Our findings also indicate that the parental home drinking environment had a positive effect on adolescent drinking over time. Overall, our findings suggest in the importance of disentangling the effects of friendship networks and family contexts when trying to understand the co-evolution of adolescent friendship tie choice and alcohol use. Future studies should further explore the risk and protective aspects of these peer and parental environments for adolescent alcohol use.

## Supporting Information

S1 Fig GOF Testing of SAB Model 1 for Jefferson High. (TIF)

S2 Fig GOF Testing of SAB Model 1 for 12 small schools. (TIF)

S1 File School contexts with key drinking effects. (PDF)

S2 File Goodness-of-Fit (GOF) Testing. (PDF)

S1 Table Stochastic Actor-Based model of friendship tie choice and adolescent drinking behavior, for 12 small schools (*n* = 1,284). (PDF)

S2 Table Ancillary models including interaction terms between school contexts and key drinking effects for 12 small schools (*n* = 1,284). (PDF)

S3 Table Distribution of drinking behavior in the twelve small schools. (PDF)
