# Supplementary figures and images for: Alcohol Use among Adolescent Youth: The Role of Friendship Networks and Family Factors in Multiple School Studies
Source: PLoS One. 2015 Mar 10;10(3):e0119965. doi: 10.1371/journal.pone.0119965 (PMC4355410; doi:10.1371/journal.pone.0119965)

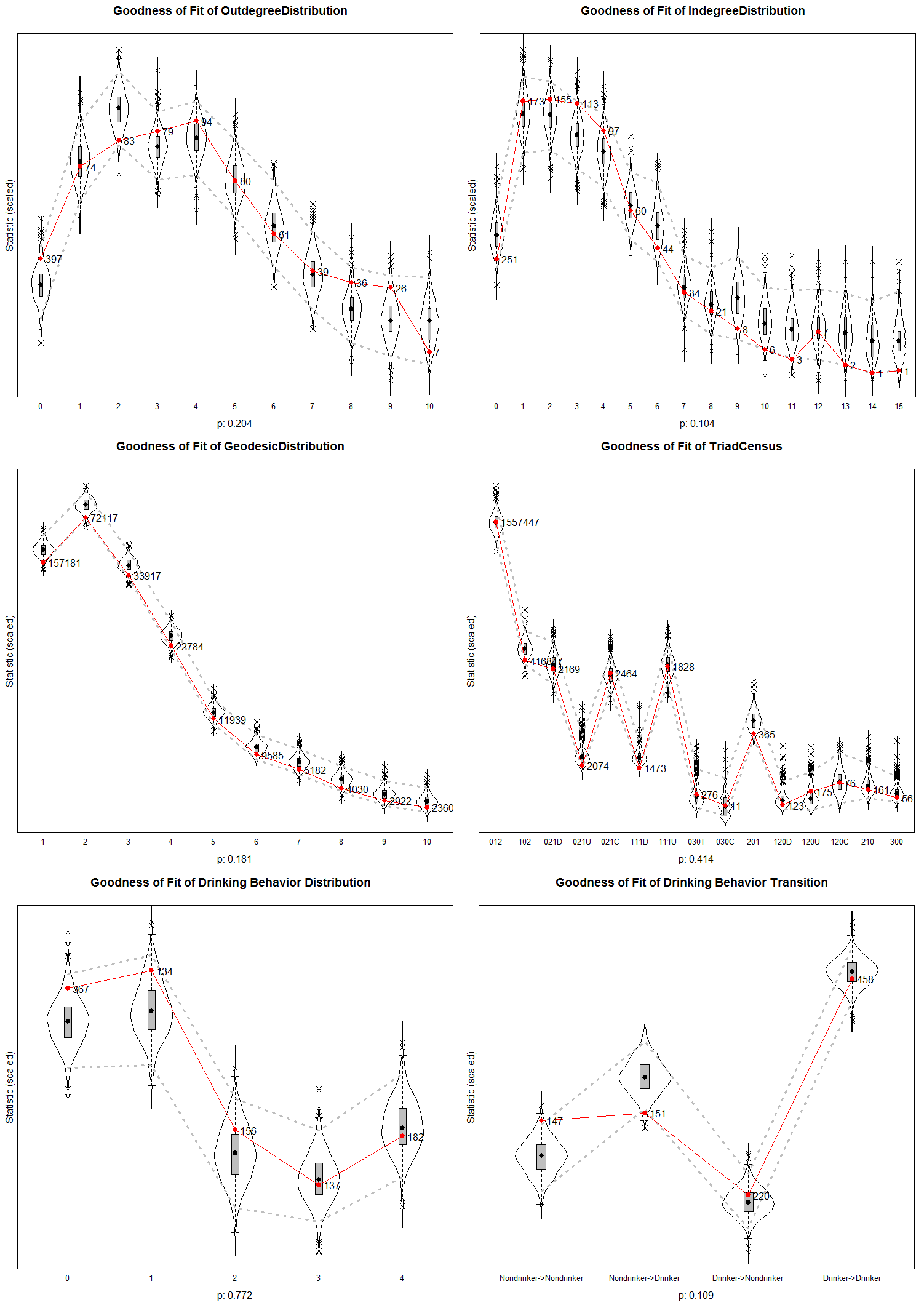

Supplement: S1 Fig — (TIF) [file pone.0119965.s001.tif]

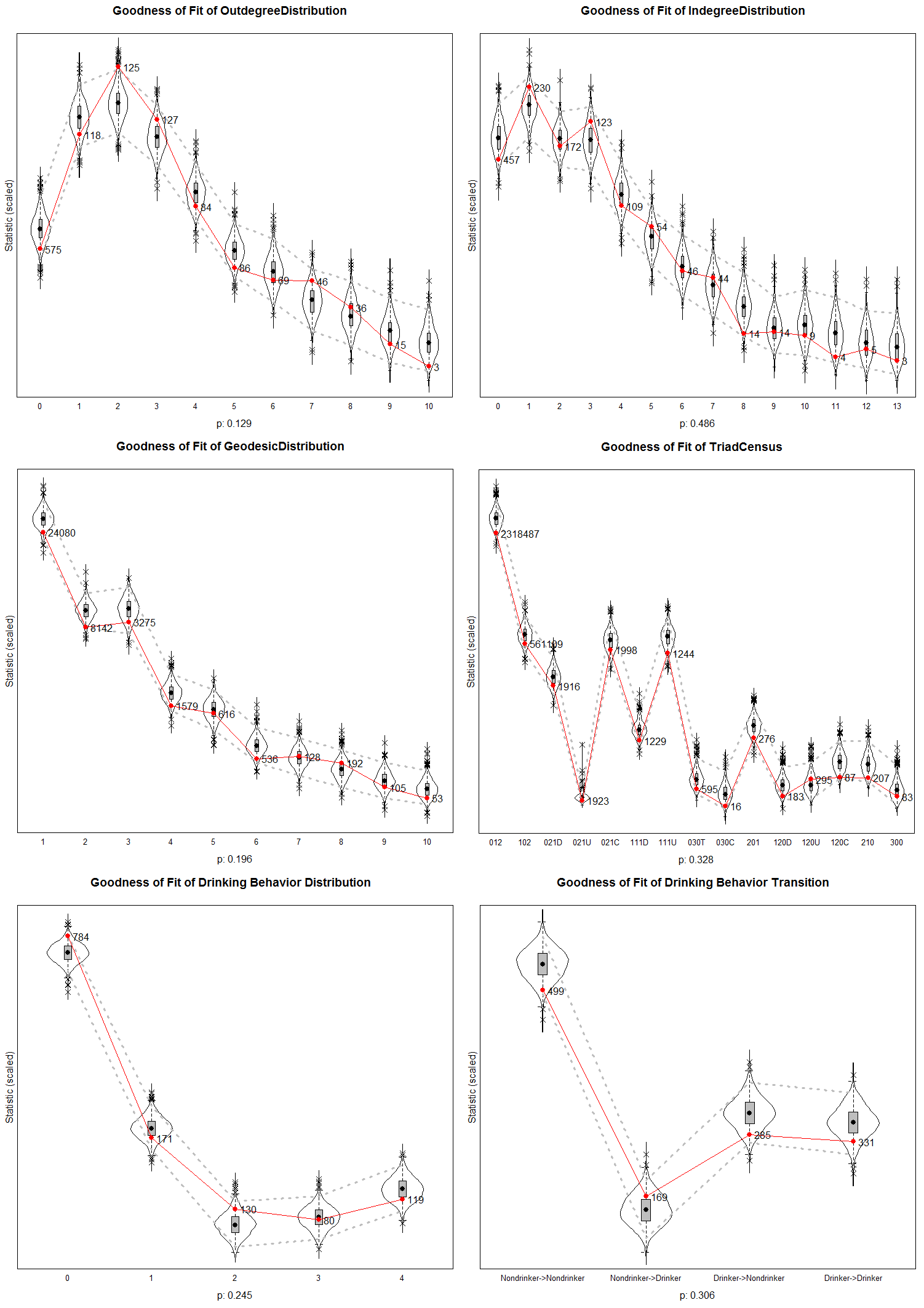

Supplement: S2 Fig — (TIF) [file pone.0119965.s002.tif]
