# Supplementary material for: Alcohol Use among Adolescent Youth: The Role of Friendship Networks and Family Factors in Multiple School Studies
Source: PLoS One. 2015 Mar 10;10(3):e0119965. doi: 10.1371/journal.pone.0119965 (PMC4355410; doi:10.1371/journal.pone.0119965)
Supplement: S1 File — (PDF) [file pone.0119965.s003.pdf]

S1 File. School contexts with key drinking effects.

To account for the possible variation in primary ecological contexts (Crosnoe, 2011) across the twelve small schools, we also estimated ancillary models along several key dimensions: (1) suburban, urban, vs. rural schools, (2) private vs. public schools, and (3) single race vs. multiple race schools. In the ancillary models, the former class in each dimension is treated as the reference group. As shown in S2 Table, the parameters were quite similar in these separate models and the interaction effects between dummy variables for these contextual variables with key drinking effects are not significant, suggesting that co-evolution of friendship tie choice and drinking behavior was similar across different types of schools.
