# Supplementary material for: Alcohol Use among Adolescent Youth: The Role of Friendship Networks and Family Factors in Multiple School Studies
Source: PLoS One. 2015 Mar 10;10(3):e0119965. doi: 10.1371/journal.pone.0119965 (PMC4355410; doi:10.1371/journal.pone.0119965)
Supplement: S2 File — (PDF) [file pone.0119965.s004.pdf]

S2 File. Goodness-of-Fit (GOF) Testing.

Goodness-of-Fit (GOF) testing has gradually become the new standard in the field of Stochastic Actor-Based modeling (Snijders, 2014). Lospinoso (2012) proposed to use the Monte Carlo Mahalanobis Distance test for Stochastic Actor-Based models, which is implemented in the package of 'RSienaTest' (Ripley et al., 2014). A *p*-value arising from the test is reported to test whether the average values of the auxiliary statistics – e.g., distribution of in-degree, out-degree, geodesic distance, and triad census – over *m* (1000 by default) simulation runs are close to the values observed in the data. Plotting functions can be used to diagnose bad fit (see more detailed in Lospinoso and Snijders, 2011, and Lospinoso, 2012).

The results for GOF testing of friendship networks and drinking behavior in Jefferson High and twelve small schools were shown in S1 and S2 Figs., respectively. In both samples, the *p*-values of the four network auxiliary statistics – the out-degree distribution, the in-degree distribution, the geodesic distance distribution, and the triad census, and those of the two behavior auxiliary statistics – behavior distribution and behavior transition, are greater than 0.05. In other words, the null hypothesis that Model 1 could reproduce several key network and behavior statistics is not rejected.

References

Lospinoso, J. A. 2012. *Statistical Models for Social Network Dynamics*. PhD thesis, University of Oxford, U.K.

Lospinoso, J. A., & Snijders, T. A. B. 2011. Goodness of fit for social network dynamics. Presentation at the Sunbelt XXXI on February 12, 2011, St. Pete's Beach, Florida.

Ripley, R. M., Snijders, T. A. B., Boda, Z., Vörös, A., & Preciado, P. (2014). *Manual for SIENA version 4.0 (version June 26, 2014)*. Available from http://www.stats.ox.ac.uk/siena/.

Snijders, T. A. B. 2014. Siena Advanced Users' Meeting 2014. Presentation at the Sunbelt XXXIV on February 19, 2014, St. Petersberg, Florida.
