# Supplementary material for: Alcohol Use among Adolescent Youth: The Role of Friendship Networks and Family Factors in Multiple School Studies
Source: PLoS One. 2015 Mar 10;10(3):e0119965. doi: 10.1371/journal.pone.0119965 (PMC4355410; doi:10.1371/journal.pone.0119965)
Supplement: S1 Table — (PDF) [file pone.0119965.s005.pdf]

S1 Table. Stochastic Actor-Based model of friendship tie choice and adolescent drinking behavior, for 12 small schools (*n* = 1,284).

Effect name	Model 1		Model 2		Model 3		Model 4	
Network decision: Friendship tie choice	beta	s.e.	beta	s.e.	beta	s.e.	beta	s.e.
Constant friendship rate (period 1)	15.72***	0.54	15.78***	0.54	15.57***	0.69	15.56***	0.76
Out-degree (density)	-2.09***	0.32	-2.03***	0.24	-2.01***	0.16	-1.89***	0.24
Reciprocity	1.79***	0.15	1.78***	0.08	1.78***	0.10	1.79***	0.09
Transitive triplets	0.23***	0.03	0.23***	0.03	0.23***	0.03	0.22***	0.03
3-cycles	-0.14**	0.05	-0.15*	0.07	-0.14*	0.05	-0.14*	0.06
In-degree popularity	0.07***	0.01	0.07***	0.01	0.07***	0.01	0.07***	0.01
In-in degree^(1/2) assortativity	-0.07**	0.02	-0.07*	0.03	-0.07*	0.03	-0.07*	0.03
Gender similarity	0.20***	0.03	0.20***	0.03	0.20***	0.04	0.20***	0.04
Parental education similarity	0.03	0.03	0.04	0.04	0.04	0.03	0.04	0.02
Grade similarity	0.45***	0.03	0.44***	0.02	0.45***	0.03	0.45***	0.02
Parental support ego	0.35***	0.09	0.34*	0.14	0.35***	0.11	0.37***	0.10
Parental monitoring ego	0.07	0.20	0.02	0.19	-0.42	0.29	0.06	0.20
Parental home drinking environment ego	-0.03	0.05	-0.04	0.08	-0.04	0.03	-0.23***	0.06
Drinking alter	0.28*	0.12	0.24***	0.05	0.29***	0.07	0.09†	0.05
Drinking ego	-0.07	0.11	-0.05	0.05	-0.06	0.06	-0.05	0.07
Drinking similarity	0.33**	0.11	0.26***	0.06	0.35***	0.06	0.29***	0.09
Parental support ego x Drinking alter			-0.17*	0.07				
Parental monitoring ego x Drinking alter					0.30	0.31		
Parental home drinking environment ego x Drinking alter							0.18*	0.06
Behavior decision: Alcohol Use								
Rate drinking behavior (period 1)	24.03***	2.46	7.37***	1.03	21.10***	1.29	23.22***	2.29
Drinking behavior linear shape	-1.78***	0.13	-4.60**	1.44	-1.75***	0.23	-1.82***	0.30
Drinking behavior quadratic shape	0.30***	0.02	0.22***	0.06	0.29***	0.02	0.30***	0.02
Drinking behavior in-degree	0.01	0.01	0.07	0.05	0.02†	0.01	0.01†	0.01
Drinking behavior peer influence	0.22*	0.10	-0.99	1.45	0.32*	0.15	0.10	0.17
Effect from gender (female=1)	-0.06	0.05	-0.40†	0.24	-0.06	0.05	-0.06	0.04
Effect from grade	0.03*	0.01	0.22*	0.09	0.03†	0.02	0.03†	0.02

Effect from depressive symptoms	0.00	0.05	0.12	0.36	0.00	0.05	0.00	0.05
Effect from parental home drinking environment	0.10***	0.03	0.53***	0.20	0.11***	0.03	0.09*	0.04
Effect from parental support	-0.01	0.07	6.21*	2.94	-0.01	0.09	-0.01	0.11
Effect from parental monitoring	-0.39*	0.18	-1.84*	0.84	-0.10	0.26	-0.39*	0.19
Effect from parental support x peer influence			23.64**	8.43				
Effect from parental monitoring x peer influence					3.08†	1.84		
Effect from parental home drinking environment x peer influence							-0.11	0.17

† Two-sided p<0.1; * Two-sided p<0.05; ** Two-sided p<0.01; *** Two-sided p<0.001
