# Supplementary material for: Alcohol Use among Adolescent Youth: The Role of Friendship Networks and Family Factors in Multiple School Studies
Source: PLoS One. 2015 Mar 10;10(3):e0119965. doi: 10.1371/journal.pone.0119965 (PMC4355410; doi:10.1371/journal.pone.0119965)
Supplement: S2 Table — (PDF) [file pone.0119965.s006.pdf]

S2 Table. Ancillary models including interaction terms between school contexts and key drinking effects for 12 small schools (*n* = 1,284).

Effect name	Model 1		Model 2		Model 3	
Friendship Tie Choice	beta	s.e.	beta	s.e.	beta	s.e.
Constant friendship rate (period 1)	15.96***	0.61	15.51***	1.03	15.50***	0.67
Out-degree (density)	-1.76***	0.15	-2.26***	0.15	-1.89***	0.17
Reciprocity	1.73***	0.07	1.77***	0.18	1.79***	0.09
Transitive triplets	0.23***	0.03	0.23***	0.04	0.23***	0.03
3-cycles	-0.14*	0.07	-0.14*	0.06	-0.14**	0.05
In-degree - popularity	0.06***	0.01	0.06***	0.02	0.07***	0.01
In-in degree^(1/2) assortativity	-0.05*	0.02	-0.06†	0.03	-0.07**	0.02
Gender similarity	0.21***	0.04	0.21**	0.07	0.20***	0.04
Parental education similarity	0.04	0.02	0.04	0.03	0.04†	0.02
Grade similarity	0.45***	0.03	0.44***	0.03	0.45***	0.03
Parental support ego	0.32***	0.09	0.32***	0.10	0.34***	0.09
Parental monitoring ego	-0.01	0.16	0.03	0.18	0.01	0.23
Parental home drinking environment ego	-0.02	0.03	-0.02	0.04	-0.04	0.03
Drinking alter	0.12	0.09	0.55***	0.12	0.33**	0.12
Drinking ego	-0.01	0.10	-0.02	0.09	-0.05	0.08
Drinking similarity	0.28***	0.08	0.35**	0.12	0.34*	0.15
School type (public=1, private=0) ego	-0.30	0.22				
Urban school (urban=1, else=0) ego			0.46	0.42		
Rural school (rural=1, else=0) ego			0.34	0.22		
Race composition (multiple=1, single=0) ego					-0.16	0.10
School type ego x drinking alter	0.17	0.23				
Urban school x drinking alter			-0.39	0.37		
Rural school x drinking alter			-0.29	0.20		
Race composition x drinking alter					-0.05	0.09
Drinking Behavior						
Rate drinking behavior (period 1)	23.23***	1.93	20.47***	1.99	23.57***	2.01
Drinking behavior linear shape	-1.75***	0.22	-2.67**	0.97	-1.94***	0.27

Drinking behavior quadratic shape	0.30***	0.02	0.27***	0.07	0.30***	0.02
Drinking behavior in-degree	0.01	0.01	0.01	0.02	0.01	0.01
Drinking behavior new similarity	0.36*	0.15	-2.07	2.17	-0.11	0.53
Effect from gender (female=1)	-0.06	0.04	-0.08	0.06	-0.07†	0.04
Effect from grade	0.03†	0.02	0.03	0.03	0.03†	0.02
Effect from depressive symptoms	0.00	0.05	0.01	0.06	0.00	0.04
Effect from parental home drinking environment	0.09*	0.05	0.11*	0.05	0.10***	0.02
Effect from parental support	-0.01	0.08	-0.02	0.11	-0.01	0.10
Effect from parental monitoring	-0.40†	0.21	-0.49	0.33	-0.38†	0.21
Effect from school type (public=1, private=0)	-0.07	0.07				
Effect from urban school (urban=1, else=0)			1.06	1.63		
Effect from rural school (rural=1, else=0)			1.03	1.53		
Effect from race composition (multiple=1, single=0)					0.17	0.26
Effect from school type x similarity	-0.26	0.27				
Effect from urban school x similarity			2.42	2.34		
Effect from rural school x similarity			2.34	2.09		
Effect from race composition x similarity					0.35	0.63

† Two-sided p<0.1; * Two-sided p<0.05; ** Two-sided p<0.01; *** Two-sided p<0.001
