# Supplementary material for: Alcohol Use among Adolescent Youth: The Role of Friendship Networks and Family Factors in Multiple School Studies
Source: PLoS One. 2015 Mar 10;10(3):e0119965. doi: 10.1371/journal.pone.0119965 (PMC4355410; doi:10.1371/journal.pone.0119965)
Supplement: S3 Table — (PDF) [file pone.0119965.s007.pdf]

S3 Table. Distribution of drinking behavior in the twelve small schools.

	In-School Survey	wave 2 In-Home Survey	In-School Survey	wave 2 In-Home Survey	In-School Survey	wave 2 In-Home Survey
	school 002 (*n* = 78)		school 003 (*n* = 163)		school 007 (*n* = 197)	
Alcohol use (past 12 months, %)						
0 = never	71.79	74.36	41.10	50.92	40.10	49.24
1 = 1 or 2 days	16.67	8.97	23.93	20.86	18.78	13.20
2 = once a month or less (3-12 times in the past 12 months)	2.56	7.69	10.43	7.36	11.17	13.20
3 = 2 or 3 days a month	1.28	3.85	8.59	6.75	8.12	11.68
4 = more than 1 or 2 days a week	7.69	5.13	15.95	14.11	21.83	12.69
	school 008 (*n* = 149)		school 028 (*n* = 168)		school 081 (*n* = 122)	
Alcohol use (past 12 months, %)						
0 = never	38.26	48.99	51.19	67.86	73.77	73.77
1 = 1 or 2 days	24.83	17.45	25.60	11.31	12.30	9.02
2 = once a month or less (3-12 times in the past 12 months)	8.72	13.42	9.52	10.12	4.10	4.10
3 = 2 or 3 days a month	8.05	8.05	6.55	5.36	2.46	4.92
4 = more than 1 or 2 days a week	20.13	12.08	7.14	5.36	7.38	8.20
	school 088 (*n* = 104)		school 106 (*n* = 102)		school 115 (*n* = 30)	
Alcohol use (past 12 months, %)						
0 = never	44.23	56.73	61.76	70.59	50.00	53.33
1 = 1 or 2 days	25.96	11.54	24.51	11.76	30.00	13.33
2 = once a month or less (3-12 times in the past 12 months)	8.65	14.42	5.88	12.75	6.67	16.67
3 = 2 or 3 days a month	8.65	4.81	4.90	1.96	13.33	6.67
4 = more than 1 or 2 days a week	12.50	12.50	2.94	2.94	50.00	10.00
	school 126 (*n* = 62)		school 194 (*n* = 46)		school 369 (*n* = 63)	
Alcohol use (past 12 months, %)						
0 = never	66.13	69.35	52.17	71.74	69.84	73.02
1 = 1 or 2 days	20.97	14.52	26.09	10.87	22.22	9.52

2 = once a month or less (3-12 times in the past 12 months)	4.84	8.06	13.04	6.52	1.59	4.76
3 = 2 or 3 days a month	3.23	1.61	2.17	4.35	1.59	6.35
4 = more than 1 or 2 days a week	4.84	6.45	6.52	6.52	4.76	6.35
